# Valorizing Coffee Silverskin Based on Its Phytochemicals and Antidiabetic Potential: From Lab to a Pilot Scale

**DOI:** 10.3390/foods11121671

**Published:** 2022-06-07

**Authors:** Juliana A. Barreto Peixoto, Nelson Andrade, Susana Machado, Anabela S. G. Costa, Helder Puga, Maria Beatriz P. P. Oliveira, Fátima Martel, Rita C. Alves

**Affiliations:** 1 REQUIMTE/LAQV, Department of Chemical Sciences, Faculty of Pharmacy, University of Porto, R. Jorge Viterbo Ferreira, 4050-313 Porto, Portugal; jpeixoto@ff.up.pt (J.A.B.P.); nelsonandrade@outlook.com (N.A.); su_tche@hotmail.com (S.M.); acosta@ff.up.pt (A.S.G.C.); beatoliv@ff.up.pt (M.B.P.P.O.); 2 Unit of Biochemistry, Department of Biomedicine, Faculty of Medicine, University of Porto, Al. Prof. Hernâni Monteiro, 4200-319 Porto, Portugal; fmartel@med.up.pt; 3 CMEMS-UMinho, Department of Mechanical Engineering, University of Minho, Campus de Azurém, 4800-058 Guimarães, Portugal; helderpuga@gmail.com; 4 Instituto de Investigação e Inovação em Saúde (I3S), University of Porto, R. Alfredo Allen, 208, 4200-135 Porto, Portugal

**Keywords:** coffee by-product, sustainability, health benefits, innovative applications, scale-up, green technology

## Abstract

This study investigates the possibility of valorizing coffee silverskin through the recovery of its bioactive compounds using a sustainable extraction method that could be industrially applied. For that, aqueous extracts were prepared using ultrasonic-assisted extraction (laboratorial scale) and, for comparison, a scale-up of the process was developed using the Multi-frequency Multimode Modulated technology. A concentration procedure at the pilot scale was also tested. The three types of extracts obtained were characterized regarding caffeine and chlorogenic acids contents, and the effects on intestinal glucose and fructose uptake (including sugar transporters expression) in human intestinal epithelial (Caco-2) cells were ascertained. The phytochemical contents of the extracts prepared at the laboratory and pilot scale were comparable (caffeine: 27.7 vs. 29.6 mg/g freeze-dried extract; 3-, 4-, and 5-caffeoylquinic acids: 0.19 vs. 0.31, 0.15 vs. 0.42, and 1.04 vs. 1.98 mg/g, respectively; 4- and 5- feruloylquinic acids: 0.39 vs. 0.43 and 1.05 vs. 1.32 mg/g, respectively). Slight differences were noticed according to the extracts preparation steps, but in general, all the extracts promoted significant inhibitions of [1,2-^3^H(N)]-deoxy-D-glucose and ^14^C-D-fructose uptake, which resulted mainly from a decrease on the facilitative glucose transporter 2 (GLUT2) and sodium-glucose linked transporter 1 (SGLT1) genes expression but not on the expression of the facilitative glucose transporter 5 (GLUT5) gene. Moreover, a synergistic effect of caffeine and 5-caffeoylquinic acid on sugars uptake was found. The results clearly show that the Multi-frequency Multimode Modulated technology is a viable option to be applied at an industrial level to recover bioactive components from silverskin and obtain extracts with antidiabetic potential that could be used to develop functional food products or dietary supplements.

## 1. Introduction

The increasing popularity and production of coffee in the last years led to a rise in the amount of wastes discarded along the coffee chain, which represents a serious environmental problem [1,2,3]. Coffee silverskin is the major by-product of coffee roasting, holding a high potential to be used/recycled for different applications (e.g., for food or cosmetic fields). This would increase its value and contribute to the sustainability and circular economy of the coffee industry. In fact, silverskin is an important source of bioactive compounds, including caffeine (0.8–1.25%), phenolics (mainly chlorogenic acids (CGAs, 1–6%)), melanoidins (17–23%), vitamins (namely vitamin E (4.2–16.8 mg/100 g), vitamin B_2_ (≈0.02 mg/100 g), and vitamin B_3_ (0.25–0.31 mg/100 g)), and minerals (8% of ash, mainly potassium (2.1–5%), magnesium (0.3–2%), and calcium (0.5–0.9%)) [1,4,5]. In addition, silverskin presents high amounts of protein (19%) and dietary fiber (56–62%), mainly insoluble (87% of total), which is also associated to several potential health benefits (e.g., prevention of cardiovascular diseases and type 2 diabetes mellitus (DM2), modulation of cholesterol, and modulation of microbiota) [1,6].

Of all the above-mentioned bioactive compounds, several studies have appointed CGAs as some of the major responsible for the positive modulation of coffee on glucose metabolism, particularly (but not only) due to their ability to inhibit intestinal glucose transporters [6,7,8,9]. The maintenance of glucose homeostasis is, indeed, of crucial importance to human physiology, and the dysregulation of this mechanism is known to be on the basis of metabolic syndrome (MetS) development [10]. MetS consists of a set of interconnected biochemical, metabolic, and clinical factors, increasing susceptibility to illnesses such as DM2, non-alcoholic fatty liver disease, and cardiovascular disease [11,12]. Although not nutritionally essential, food-derived glucose plays an important role in the regulation of postprandial glycemia. Plasma glucose levels, in turn, not only play a role in the control of food intake but also regulate insulin secretion, and peripheral insulin resistance (with its associated glucose intolerance) is closely linked to the development of MetS [10,13]. Additionally, several dietary intervention studies, with epidemiological and clinical data, have suggested the crucial role of fructose, which is present in sucrose and high fructose corn syrup (HFCS), in the MetS epidemic [14,15].

Furthermore, caffeine has also been widely associated with numerous physiological effects, including those related to MetS. However, the real impact (beneficial or harmful) of caffeine in factors involved in the pathogenesis of MetS is still quite controversial. While, on the one hand, several studies are showing that acute consumption of caffeine decreases glucose tolerance (e.g., by increasing glycogenolysis and inhibiting muscle glucose uptake) and increases insulin resistance and arterial blood pressure [16,17,18,19], on the other hand, numerous studies present opposite conclusions, demonstrating that the chronic consumption of caffeine decreases impaired glucose tolerance, insulin resistance, hypertension, body weight, and visceral fat (being these last two effects related to a stimulation of lipolysis, an increase in cellular thermogenesis, energy consumption, and metabolic rate, and a decrease in lipogenesis) [16,20,21,22]. Therefore, in recent years, many authors have defended that the effects of caffeine seem to be related to the acute vs. chronic exposure to this compound, as it has been shown that the harmful effects found in the acute consumption of caffeine disappear and become protective when caffeine is chronically consumed [16]. However, more studies are still needed to prove this hypothesis.

Despite the richness of coffee silverskin in the mentioned bioactive compounds [1,3,4] and their potential role in the prevention of health disorders (namely metabolic ones), the current strategies still remain in dispatching this by-product to landfills or using it as firelighters [1]. Nonetheless, in the last years, the scientific community has invested many efforts in studying the possibility of using coffee silverskin or their extracts in foods formulation. For example, some studies have tested their incorporation in beverages, biscuits, bread, yogurts, etc. [23,24]. Some of them are already patented [24], but as far as we know, none is available in the market for now.

In addition, the recovery of bioactive compounds from silverskin has also been a target of study due to the need to find greener extraction methods as an alternative to the conventional ones, which are dangerous to the environment (due to the use of organic solvents), and they are time and energy consuming [3,25]. Some green extraction techniques have been proposed, namely ultrasound-assisted extraction (UAE), appointed as a green, fast, economic, and efficient technique to recover bioactive compounds from natural matrices [3,26]. Nonetheless, although very promising, the studies applying this technique to obtain bioactive compounds from coffee silverskin are still relatively scarce in the literature [23], and to the best of our knowledge, it has not been implemented at a larger scale. Bearing this in mind, we wondered if it would be possible to scale up this sustainable methodology, having in view the valorization of this by-product, with a viable economic return for industry, simultaneously contributing to health maintenance. Therefore, the novelty of this work remains in studying the possibility of scaling up the UAE to recover bioactive compounds with antidiabetic potential from silverskin. For that, besides preparing the extracts at a laboratory scale, a pilot scale of the extracting process was developed to ascertain the effectiveness and reproducibility of the method to be implemented on a larger scale (industrial scale). In addition, an enrichment step at the pilot scale was also performed to test the possibility of concentrating the bioactive compounds. After preparation, the extracts were characterized in terms of bioactive composition, and their effects on intestinal glucose and fructose uptake and on sugar transporters expression using human intestinal epithelial (Caco-2) cells were evaluated. In addition, to assess the contribution of the two major bioactive compounds identified in the extracts, their effects on intestinal sugar uptake were also evaluated, individually and combined, at the concentrations present in the extracts. As far as we know, this is the first study that scales up the extraction process of silverskin compounds by an ultrasonic-assisted procedure and that assesses the effect of silverskin on intestinal glucose and fructose uptake, as well as the effects of caffeine and 5-caffeoylquinic acid (5-CQA), individually and combined, on the intestinal fructose uptake.

## 2. Materials and Methods

### 2.1. Reagents and Standards

The standards 5-caffeoylquinic acid (5-CQA), 4-CQA, 3-CQA, 5-feruloylquinic acid (5-FQA), 4-FQA, and caffeine, as well as glacial acetic acid and HPLC grade methanol, were all from Sigma-Aldrich (St. Louis, MO, USA).

For the cellular assays, all chemicals were obtained from standard commercial suppliers and were of analytical grade quality. MEM medium, HEPES (N-2-hydroxyethylpiperazine-N′-2-ethanesulfonic acid), antibiotic/antimycotic solution (100 U/mL penicillin; 100 mg/mL streptomycin and 0.25 mg/mL amphotericin B), trypsin–EDTA (ethylenediamine tetra-acetic acid) solution, NADH (reduced nicotinamide adenine dinucleotide), sulforhodamine B (SRB), sodium pyruvate, sodium salt, and trichloroacetic acid were all purchased from Sigma (St. Louis, MO, USA). Fetal calf serum was from Invitrogen Corporation (Carlsbad, CA, USA). DMSO and Triton X-100 were from Merck (Darmstadt, Germany). ^14^C-D-Fructose (fructose, D-[^14^C(U)]; (^14^C-FRU); specific activity 250–360 mCi/mmol) and [1,2-^3^H(N)]-deoxy-D-glucose ((^3^H-DG); specific activity 60 mCi/mmol) were acquired from American Radiolabeled Chemicals (St. Louis, MO, USA). NZYol reagent was purchased from NZYTech (Lisbon, Portugal), qScript cDNA SuperMix was obtained from Quanta Biosciences (Gaithersburg, MD, USA), and KAPA SYBRw FAST qPCR Master Mix was from Kapa Biosystems (Wilmington, MA, USA).

Ultrapure water was obtained in a Milli-Q water purification system (Millipore, Bedford, MA, USA).

### 2.2. Samples

Coffee silverskin, obtained after roasting commercial coffee blends comprising mixtures of both arabica (*Coffea arabica*) and robusta (*Coffea canephora*) species (210 °C, 10 min), was kindly provided by BICAFÉ, Torrefação e Comércio de Café, Lda. (Portugal). The sample was stored at room temperature in a dry place and protected from light until extracts preparation.

### 2.3. Extracts Preparation by Ultrasound-Assisted Extraction

#### 2.3.1. Laboratorial Scale

**Figure 1:**
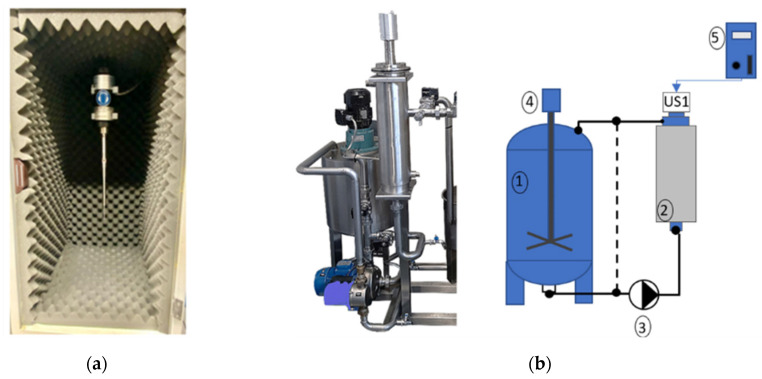

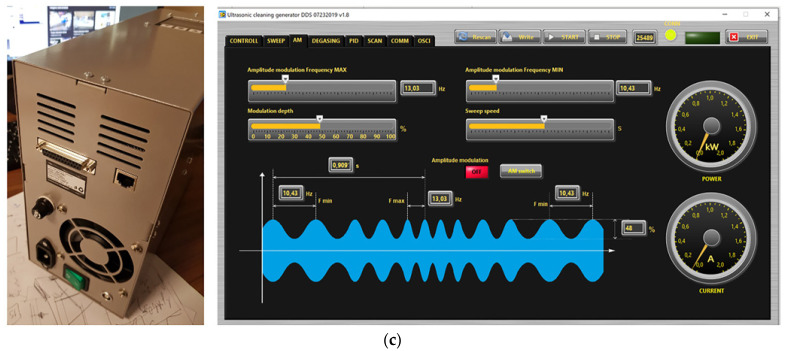
Ultrasonic caffeine extraction: (**a**) Laboratorial scale; (**b**) Pilot scale (1—Tank, 2—Chamber of extraction/homogenization, 3—Pump, 4—Mixer, 5—MMM Generator); (**c**) MPI Generator and software controller.

The extracts were prepared at a laboratory scale using a SONOPULS ultrasonic homogenizer HD4050 (BANDELIN electronic GmbH & Co. KG, Heinrichstrasse, Germany) composed of an ultrasonic generator, converter of 20 kHz, and a probe with a cylindrical shape (Figure 1a). The whole system has been operated independently from the applied load with a resonant frequency of 20 kHz and a constant amplitude. A feedback capability of the ultrasonic device used ensures that the maximum acoustic power allowed is not exceeded.

The extractions were performed, in octuplicate, using a rigorous amount (0.500 g) of ground sample and 25 mL of distilled water. Extractions were performed under the following conditions, based on a previous study [3]: extraction time of 10 min; resonant frequency of 20 ± 0.5 kHz, and constant amplitude of 50% of the system’s capacity. At the end of the 8 extractions, the replicates were combined, filtered, and freeze-dried (−80 °C, 0.015 mbar; TELSTAR, Cryodos freeze dryer, Barcelona, Spain). Aliquots of this extract (S_LS) were analyzed by chromatography (Section 2.4) immediately after preparation (in liquid form) and after lyophilization. The freeze-dried samples were used for cellular assays and prepared as described in Section 2.5.

#### 2.3.2. Pilot Scale

The extracting experiments carried out at pilot scale were conducted using a Multi-frequency Multimode Modulation (MMM) technology consisting of a high-power ultrasonic converter, a booster, an acoustic waveguide, and a radiator. The ultrasonic device presents a sweeping-frequency capability to adaptively modulate waveform generated by an MMM ultrasonic generator. The equipment was fully controlled through a dedicated software developed by MPI (Le Locle, Switzerland). With the implemented feedback loop, the most efficient ultrasonic parameters for the selected resonant frequency and electric power were adjusted in order to produce the highest amplitude and largest frequency spectrum on the medium. The ultrasonic device was composed of a booster with amplification of 1:2.5, a holed waveguide (diameter of 35 mm) and a holed cylindrical shape radiator with length and diameter of 397 mm and 45 mm, respectively. Furthermore, the ultrasonic extraction process was run by sending periodical ultrasonic pulse trains (with defined ON and OFF time intervals), thus, combining relaxation with time-evolving process transients between ON periods. The MMM generator and converter were optimized to work at 19.80 ± 0.10 kHz with an amplitude of 40%, according to Puga et al. [3].

**Figure 2:**
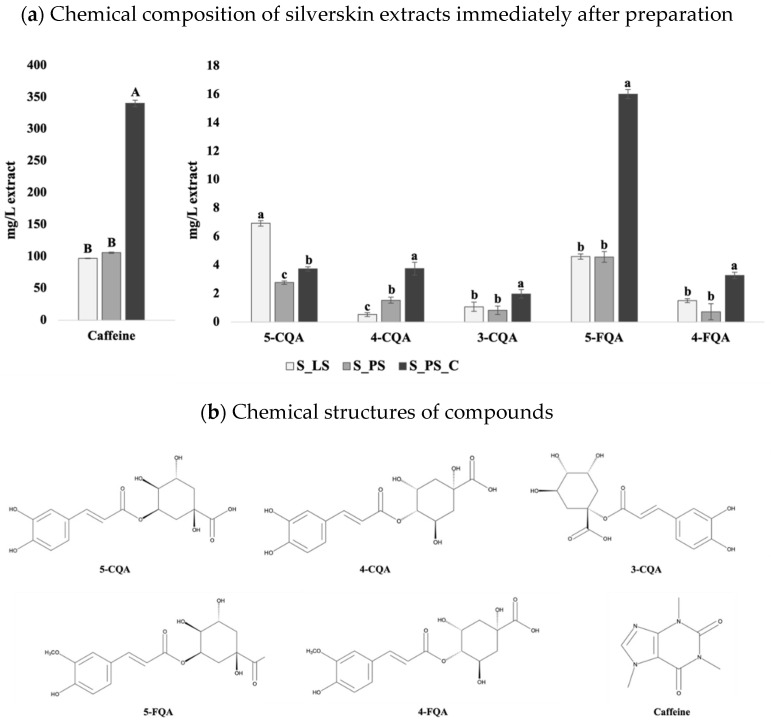
(**a**) Comparison of the caffeine and chlorogenic acids contents of silverskin extracts (expressed in mg/L of extract) prepared under different conditions (S_LS: laboratorial scale; S_PS: pilot scale; S_PS_C: pilot scale_ concentrated). The samples were analyzed immediately after preparation. Different letters for each compound denote significant differences between samples at *p* < 0.05. (**b**) Chemical structures of caffeine, 5-CQA, 4-CQA, 3-CQA, 5-FQA, and 4-FQA. CQA, caffeoylquinic acid; FQA, feruloylquinic acid.

Based on the silverskin/water ratio used at the laboratorial scale (Section 2.3.1), 5 kg of silverskin was mixed with 250 L of water in a tank and then recirculated in the system (Figure 1b) for approximately 10 min. The pump was activated, and the homogenized mixture was driven through the chambers containing the ultrasonic devices and, finally, for the tank again. In the chamber (Figure 1b-2), the transducers coupled to a cylinder sonorode were working during all the extraction process at ON (3 s)–OFF (3 s) mode. At the end of the process, the S_PS extract was obtained. Aliquots of this extract were analyzed by chromatography (Section 2.4) immediately after preparation (in liquid form) and after lyophilization. The freeze-dried sample was used for cellular assays and prepared as described in Section 2.5.

To obtain the concentrated extract (S_PC_C), similar conditions to those described for S_PS were performed. However, after extraction, the mixture was passed through a 1 mm mesh filter (placed in the top of the tank), separating the extract from the solid residue, being this last rejected. The filtered extract in the system was then mixed with a new sample of silverskin (5 kg), and the system recirculated again according to the above-described conditions. This was repeated two more times, and after 4 subsequent cycles, the concentrated extract (S_PC_C) was obtained. Aliquots of this extract were analyzed by chromatography (Section 2.4) immediately after preparation (in liquid form). After that, a 3 L aliquot was filtered, concentrated in a rotatory evaporator (at 40 °C) until 300 mL (process that lasted 8 h), and then freeze-dried (−80 °C, 0.015 mbar; TELSTAR, Cryodos freeze dryer, Barcelona, Spain). The freeze-dried sample was analyzed by chromatography (Section 2.4) and used for cellular assays (Section 2.5).

### 2.4. Chlorogenic Acids Profile and Caffeine Content by RP-HPLC-DAD

For chromatographic analyses of liquid extracts (before lyophilization), a sample aliquot (1 mL) was diluted with deionized water (1:10). In the case of freeze-dried extracts, a rigorous amount (≈10.00 mg) of powder (previously homogenized) was redissolved in 10 mL of deionized water. Each sample was prepared in triplicate.

Samples were then centrifuged (4500 rpm, 10 min; Haraeus Sepatech Biofuge Pico, Heraeus Instruments, Germany). The supernatant was collected into Eppendorfs and further centrifuged (13,000 rpm, 10 min; Heraeus Fresco 17 Centrifuge, Thermo Fisher Scientific, Germany). The new supernatant was transferred into injection vials and analyzed by RP-HPLC-DAD according to the conditions described by Puga et al. [3]. The chlorogenic acids were monitored at 320 nm and caffeine was monitored at 274 nm. The compounds were identified by comparing retention times, elution orders, and UV absorption spectra with authentic standards. Chromatographic data were analyzed with Borwin-PDA Controller Software from JMBS Developments (Le Fontanil, France).

### 2.5. Cellular Assays

#### 2.5.1. Caco-2 Cell Culture

The Caco-2 cell line was obtained from ATCC (Manassas, VA, USA) and was used between passage numbers 8 and 18. The cells were grown in Minimum Essential Medium (Sigma, St. Louis, MO, USA) containing 5.55 mM glucose and supplemented with 15% fetal calf serum, 25 mM HEPES, 100 units/mL penicillin, 100 μg/mL streptomycin, and 0.25 μg/mL amphotericin B, in a humidified atmosphere (5% CO_2_/95% air). Cells were cultured in plastic culture dishes (21 cm^2^; ∅ 60 mm; Corning Costar, Corning, NY, USA) with culture medium change every 3–4 days and culture split 1:3 (0.25% trypsin-EDTA, 5 min, 37 °C) every 10 days.

For the experiments, Caco-2 cells were seeded on 24-well plastic cell culture clusters (2 cm^2^; ∅ 16 mm; Corning Costar) and used at 100% confluence (10 days after the initial seeding). Then, 24 h before the experiments, the culture medium was made free of fetal bovine serum.

#### 2.5.2. Extracts and Standards Preparation

For the cellular assays, a rigorous amount (≈10.00 mg) of each freeze-dried extract (previously homogenized) was dissolved in 100 μL of distilled water, resulting in a concentrated extract (100 mg/mL). Then, in each experiment, different extract concentrations were prepared by dissolving an aliquot of the concentrated extract in culture medium or glucose-free Krebs buffer.

In each experiment, controls were run using water. For the quantification of ^3^H-DG and ^14^C-FRU uptake, the extracts were tested at different concentrations (0.01, 0.1, and 1 mg/mL). For the remaining assays, the extracts were tested at 1 mg/mL. In addition, the effects of the analytical standards caffeine and 5-caffeoylquinic acid (the major compounds found in all freeze-dried extracts) were also tested, individually and mixed, on ^3^H-DG and ^14^C-FRU uptake by Caco-2 cells, at the same concentrations found in 1 mg/mL of the corresponding extracts. The compounds were dissolved in DMSO, and their controls were run in the presence of this solvent. None of the solvents used (distilled water and DMSO) significantly affected the measured parameters (^3^H-DG uptake and ^14^C-FRU, cellular viability, and proliferation; data not shown).

#### 2.5.3. Quantification of ^3^H-Deoxy-D-Glucose (^3^H-DG) and ^14^C-Fructose (^14^C-FRU) Uptake by Caco-2 Cells

For these experiments, the cells were incubated in glucose-free Krebs buffer containing 125 mM NaCl, 4.8 mM KCl, 1.2 mM MgSO_4_, 1.2 mM CaCl_2_, 25 mM NaHCO_3_, 1.6 mM KH_2_PO_4_, 0.4 mM K_2_HPO_4_, and 20 mM HEPES (pH 7.4). After removal of culture medium, the cells were washed with 0.3 mL buffer at 37 °C and then preincubated (20 min) in buffer (0.3 mL, 37 °C). Sugar uptake was initiated by adding 0.3 mL of medium at 37 °C containing ^3^H-DG (10 nM) or ^14^C-FRU (100 nM). After 6 min, incubation was stopped by removing the incubation medium, placing the cells on ice, and rinsing with 0.5 mL ice-cold buffer. The cells were then solubilized with 0.1% (*v/v*) Triton X-100 (in 5 mM Tris HCl, pH 7.4) and placed at 37 °C overnight. Radioactivity in the cells was measured by liquid scintillation counting. The extracts or compounds to be tested were present for 24 h before and after the uptake experiments (preincubation and incubation periods).

#### 2.5.4. Quantitative Real-Time Polymerase Chain Reaction (qRT-PCR)

Total RNA was isolated from Caco-2 cells treated for 24 h with compounds to be tested, using NZYol reagent (NZYTech, Lisbon, Portugal) according to manufacturer’s instructions. Total RNA was treated with DNAse I, and 1 mg of the resulting DNA-free RNA was reverse transcribed using qScript cDNA SuperMix (Quanta Biosciences, Gaithersburg, MD, USA) in 20 μl of final reaction volume, according to manufacturers’ instructions. Quantitative real-time polymerase chain reaction (qRT-PCR) was run on Lightcycler96 (Roche Applied Science, Indianapolis, ID, USA). Cycling conditions for human β-actin, SGLT1, GLUT5, GLUT2 primers were as follows: denaturation (95 °C for 5 min), amplification and quantification (95 °C for 10 s, annealing temperature (AT: 65 °C, 60 °C, 65 °C, and 59 °C, respectively) for 10 s and 72 °C for 10 s, with a single fluorescence measurement at the end of the 72 °C for 10 s segment) repeated 45 times; a melting curve program ((AT + 10) °C for 10 s and 95 °C with a heating rate of 0.1 °C/s and continuous fluorescence measurement) and a cooling step to 37 °C for 30 s. The amount of SGLT1, GLUT5 and GLUT2 mRNA was normalized to the amount of human β-actin mRNA (housekeeping gene). The primer pairs used were: β-actin: 5′-AGA GCC TCG CCT TTG CCG AT-3′ (forward) and 5′-CCA TCA CGC CCT GGT GCC T-3′ (reverse). SGLT1: 5′-TGG CAA TCA CTG CCC TTT A-3′ (forward) and 5′-TGC AAG GTG TCC GTG TAA AT-3′ (reverse). GLUT2: 5′-CAG GAC TAT ATT GTG GGC TAA-3′ (forward) and 5′-CTG ATG AAA AGT GCC AAG T-3′ (reverse). GLUT5: 5′-ACC GTG TCC ATG TTT CCA TT-3′ (forward) and 5′-ATT AAG ATC GCA GGC ACG AT-3′ (reverse). Data were analyzed using LightCycler^®^ 96 SW 1.1 analysis software (Roche, Mannheim, Germany), and results were analyzed by the Ct method [27].

#### 2.5.5. Determination of Cell Viability and Culture Mass

In order to rule out the possibility that the effects of silverskin extracts on sugar uptake are the result of a cytotoxic effect, two additional experiments were performed: evaluation of cell viability and evaluation of culture mass.

At the end of the 24 h exposure to the extracts, cell viability was assessed by quantification of extracellular LDH activity, as previously described [28].

At the end of the 24 h exposure to the extracts, cell culture mass was determined by the sulforhodamine B (SRB) assay, which reports on intracellular protein content, as previously described by Andrade et al. [28].

#### 2.5.6. Total Protein Determination

The protein content of cell monolayers was determined as described by Bradford [29], using human serum albumin as standard.

### 2.6. Statistical Analysis

Data were expressed as mean ± standard deviation or mean ± standard error of the mean (for cellular experiments). “n” indicates the number of replicates of at least 2 independent experiments. One-way ANOVA was used to reveal significant differences between samples, followed by Duncan’s test to make pairwise comparisons between means. Moreover, statistical significance of the difference between two groups was evaluated by Student’s t-test. The level of significance for all hypothesis tests (*p*) was 0.05. Statistical treatments were carried out using the GraphPad Prism version 7.0 software (San Diego, CA, USA) and the IBM SPSS 26 for macOS (SPSS Inc., Chicago, IL, USA).

## 3. Results and Discussion

### 3.1. Chemical Characterization of the Extracts

Several studies appointed ultrasound-assisted extraction (UAE) as a sustainable and effective extraction method since it is less time- and energy-consuming, it requires less consumption of solvents (and allows the use of green and safer solvents, such as water), and it efficiently warrants the recovery of bioactive compounds from natural matrices [26,30,31]. Indeed, in a previous work, we proved that compared to an optimized solid–liquid extraction (using a hydroethanolic solvent (1:1) at 40 °C for 60 min) [32], the acoustic probe allowed a higher recovery of antioxidants from coffee silverskin, in only 10 min, using only water as extraction solvent and with no need to grind the sample [3]. In this case, direct ultrasonic agitation produces and transfers maximal ultrasonic energy to the medium. Additionally, the generation of high-amplitude agitation in the sonicated liquid gradually produces time-evolving non-linear acoustic effects (coming closer to plastic deformation effects), breaking molecular bonds and favoring the mass transfer between the substrate and the liquid medium. For all these reasons, UAE has been widely considered a viable technological option to be applied at an industrial level matrices [26,30,31], although there are still not many studies applying this extraction technique with coffee silverskin.

Bearing this in mind, in this study, coffee silverskin extracts were prepared at both laboratorial (S_LS) and pilot scales (S_PS) applying the UAE technology in order to compare the two processes and understand if a larger scale for coffee silverskin extraction could be successfully reached and implemented at an industrial level. Moreover, an attempt of extract concentration (in order to enrich the aqueous fraction in bioactive compounds) was also performed by using the same liquid extract for four subsequent extractions in cycle (S_PS_C), as described in Section 2.3.2. The different extracts were characterized immediately after their preparation regarding their composition in caffeine and chlorogenic acids, and the results are presented in Figure 2a. The chemical structures of these compounds are depicted in Figure 2b.

The results clearly show comparable values obtained from both processes (S_LS and S_PS) showing only significant differences (*p* < 0.05) in the contents of 5-CQA and 4-CQA, although the values were in the same range and near each other. The amounts of caffeine and 5-CQA herein obtained are in accordance with those reported in previous studies [1,3,33], thus validating our results and the extraction method used. The slight differences between the three extracts are probably due to some variations between the methods (in the lab, the sample was ground before extraction, while at the pilot scale, that step was omitted due to the high amounts of sample; the ultrasonic probes were different, etc.). However, the similarity of results (*p* > 0.05) found for caffeine and the remaining CGAs support the high reproducibility of the process with the scale-up. Moreover, it was also possible to concentrate the sample by performing subsequent cycles of silverskin extraction. More concretely, when comparing the concentrated extract (S_PS_C) with the non-concentrated one (S_PS), both obtained at a pilot scale, it can be seen that caffeine, 4-FQA, and 5-FQA were successfully concentrated (≈3.2 to 4.5-fold higher contents in S_PS_C than in S_PS), while 3-, 4-, and 5-CQA were concentrated in a lower extent (≈1.3 to 2.4-fold higher contents in S_PS_C than in S_PS). This might be explained due to the susceptibility of CQAs to degradation, isomerization, transesterification, or conversion into lactones [34,35]. In fact, although FQAs are also susceptible, it is reported that, for example, the formation of feruloylquinic acid lactones (FQLs) occurs much less often than caffeoylquinic acid lactones (CQLs) [34,36], thus corroborating these results. However, the concentration step was well succeeded, opening here new possibilities of direct applications of the liquid extract where higher contents of caffeine and antioxidants are required, such as for example, in the areas of food supplements for physical and cognitive improvement or, even, for cosmetics.

In order to obtain a powder to be used for further applications, the liquid extracts S_LS and S_PS presented in Figure 2 were directly subjected to lyophilization. In the case of S_PS_C, a 90% further concentration was performed using a rotatory evaporator and only after that the extract was lyophilized. Caffeine contents and CGAs profiles of the obtained powders are presented in Table 1.

Independently of the original concentration of the extract, the freeze-dried powder from the different processes always presented a similar profile/content of the analyzed compounds. In turn, the higher the concentration of the liquid extract before lyophilization, the higher the amount of freeze-dried power obtained. In this case, when lyophilizing 100 mL of S_PS extract (5 kg silverskin/250 L), 0.311 g of powder was obtained, while for S_PS_C (that used 5 kg × 4 for extraction in the same 250 L recirculating in cycles), 1.011 g was produced.

**Figure 3:**
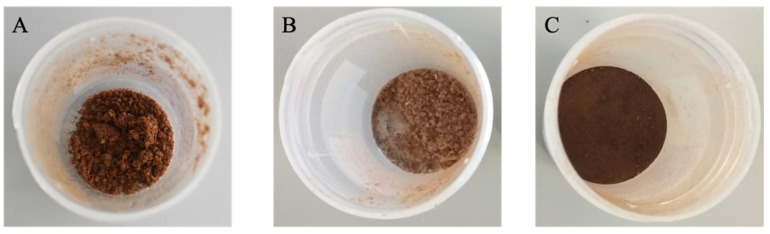
Color of the different freeze-dried extracts. (**A**) S_LS (laboratorial scale) freeze-dried extract; (**B**) S_PS (pilot scale) freeze-dried extract; (**C**) S_PS_C (pilot scale; concentrated) freeze-dried extract.

However, although the compounds’ profiles were relatively similar (in the same range of contents) between the freeze-dried extracts (Table 1), some differences were noticed. These differences were significant and notorious for 5-CQA: the freeze-dried extract prepared at laboratorial scale (S_LS) presented significantly higher contents than the extract prepared at pilot scale, but that was only subjected to one cycle of extraction (S_PS) and this, in turn, presented significantly higher contents than the freeze-dried extract prepared at pilot scale being subjected to four extraction cycles and rotatory evaporation (S_PS_C). Furthermore, 4-CQA content was higher in the S_PS powder and, although not statistically significant, the S_LS powder presented higher amounts of 3-CQA and 5-FQA than S_PS, which were also higher than those found in S_PS_C. Notwithstanding, the amounts of caffeine and 4-FQA were rather similar between all freeze-dried extracts. Considering that were mainly the CGAs amounts that differed between the powders (differences particularly clearer between S_PS_C vs. S_PS and S_LS), and taking into account the known susceptibility of CGAs to temperature when compared to caffeine (thermoresistant) [22,33,34,37,38], it is possible that the concentration procedures applied at the pilot scale might have degraded these compounds. Indeed, the step of rotatory evaporation to concentrate the liquid extract before lyophilization, although performed at a relatively low temperature (40 °C) lasted about 8 h, which might have led to some CGAs degradation. Nonetheless, as it will be discussed in further sections, although the S_PS_C powder seemed to be the poorest concerning CGAs contents, these lower amounts were not reflected in lower beneficial effects on intestinal sugar uptake capacity by Caco-2 cells, as it would be expected based on its CGAs contents. Therefore, this seems to suggest that CGAs, particularly 5-CQA, might have been converted into CGAs derivatives, such as CGA lactones (CGLs), also with recognized health benefits, including on glucose metabolism (through the enhancement of insulin secretion) [34,36,38,39]. Moreover, CGAs might also be incorporated in melanoidins through Maillard reactions, which are a complex group of compounds with several well-recognized bioactive properties [22,40,41]. In fact, the possibility of such compounds being present in S_PS_C gains even more support if we compare the colors of the different freeze-dried extracts. As can be observed in Figure 3, the powder obtained after freeze-drying the extract S_PS_C is darker than that of S_PS. Indeed, considering that melanoidins highly contribute to the brown color of foods and beverages where they are formed [40,41,42], the most brownish color of S_PS_C powder may reveal a higher concentration of melanoidins.

Notwithstanding, due to the huge variety of structures (proteins, amino acids, polysaccharides, CGAs, etc.) that might constitute melanoidins, their exact composition is not entirely known. Therefore, in further studies, it would be interesting to better understand which type of melanoidins and also CGLs might have been present in our extracts as well as to study their effects on the intestinal uptake of sugars.

### 3.2. Effect of the Different Extracts on ^3^H-DG and ^14^C-FRU Uptake

**Figure 4:**
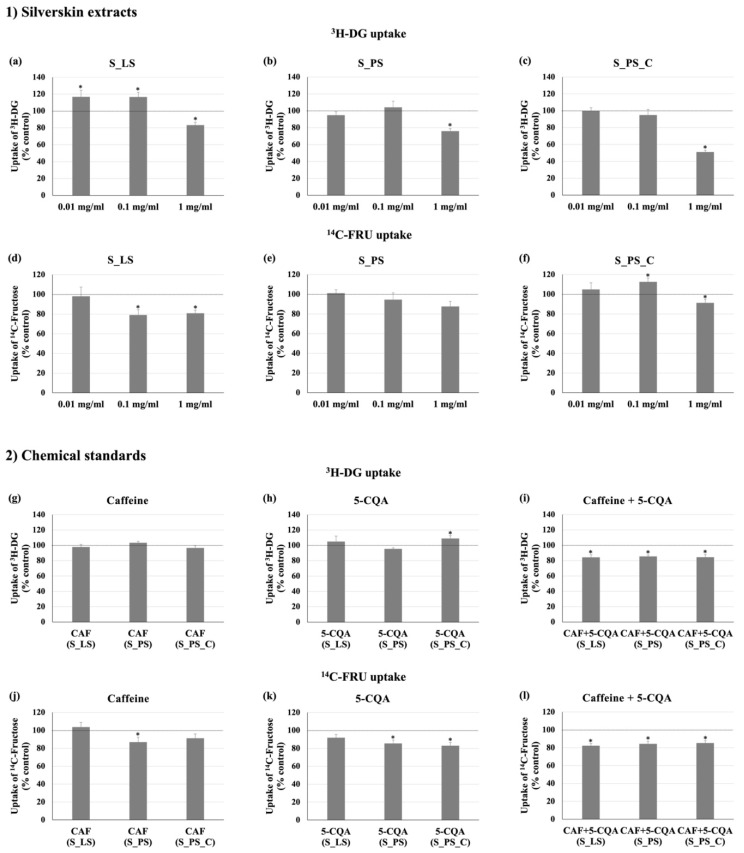
Effect of coffee silverskin freeze-dried extracts (**1**) and caffeine and 5-caffeoylquinic acid standards (**2**) on ^3^H-DG and ^14^C-FRU uptakes. Caco-2 cells were exposed to different concentrations of extracts S_LS, S_PS, and S_PS_C (**a**–**f**), in the presence of caffeine (**g**,**j**), 5-CQA (**h**,**k**), and caffeine plus 5-CQA (**i**,**l**), in the concentrations present in extracts S_LS, S_PS, and S_PS_C (g/L), or in the presence of the respective solvent (control) for 24 h. Uptake was measured by incubating cells at 37 °C with 10 nM ^3^H-DG or 100 nM ^14^C-FRU for 6 min. For each experiment, *n ≥* 6. Results expressed as mean ± SEM. *, significantly different from control at *p* < 0.05.

The influence of all silverskin extracts (S_LS, S_PS, and S_PS_C) on the intestinal absorption of glucose and fructose was studied. Although several studies report a positive effect of coffee silverskin on intestinal sugar uptake due to the presence of caffeine and CGAs [8,43], as far as we know, this is the first study that assesses the effect of this by-product on the intestinal uptake of both glucose and fructose. Regarding glucose (^3^H-DG), all the extracts, at the highest concentration tested (1 mg/mL), were able to significantly reduce its uptake by Caco-2 cells (Figure 4a–c). This was especially evident with S_PS_C (1 mg/mL), which caused a very marked (around 50%) reduction. Concerning fructose (^14^C-FRU), S_LS and S_PS_C extracts, but not S_PS extract, were able to reduce the cellular uptake of this sugar (Figure 4d–f). Interestingly, the extent of ^3^H-DG and ^14^C-FRU uptake reduction attained by each extract was different. For example, while S_PS_C was the most effective in inhibiting ^3^H-DG uptake, the S_LS extract was the most effective in reducing ^14^C-FRU uptake. Moreover, S_LS (1 mg/mL) was similarly effective in inhibiting the uptake of both sugars, while S_PS and S_PS_C (1 mg/mL) were more effective in reducing ^3^H-DG uptake. This difference may be related to the presence of several bioactive compounds in different amounts in the three extracts. First, because the distinct bioactive compounds present in the three extracts may have distinct effects on ^3^H-DG and ^14^C-FRU uptake.

Second, it is expected that these distinct compounds will interact with each other, which may also contribute to the different effects. As above discussed, the different conditions applied to extract the bioactive compounds from silverskin (e.g., laboratory vs. pilot scale and concentration of one of the extracts) originated extracts with slightly different concentrations of CGAs, as well as of other possible bioactive compounds not analyzed in this study (CGA lactones and melanoidins). Thus, considering the complex mixture of compounds present in these extracts, it is expected that they interact with each other by additive, synergic or antagonistic mechanisms at different extents, resulting in the higher or lower observed effects on the intestinal absorption of both sugars. For example, different compounds may develop stable complexes with each other with greater activity than the individual compounds, thus resulting in synergistic activities, or, on the other hand, form complexes and adducts that result in a lower activity and, hence, in antagonistic effects [44]. Therefore, in further studies, it would be interesting to better understand not only which other compounds may be present in these extracts and which may also have activity but also to study such interactions between all of them.

### 3.3. Effect of Caffeine and 5-CQA on ^3^H-DG and ^14^C-FRU Uptake

Several studies have already documented that the major bioactive compounds identified and quantified in these extracts by HPLC-DAD (caffeine and CGAs) may act positively on sugar metabolism, namely by inhibiting intestinal glucose transporters [7,8,9,16]. In order to better understand the results described in Section 3.2. and to complement the information already available in the literature, we decided to additionally study the effects of the major compounds found in our freeze-dried extracts, namely caffeine and 5-CQA (as representative of the CGAs group) on ^3^H-DG and ^14^C-FRU absorption by Caco-2 cells, at the concentrations present in 1 mg/mL of the corresponding extracts (caffeine: 2.77 × 10^−2^ mg/mL, 2.96 × 10^−2^ mg/mL and 2.73 × 10^−2^ mg/mL, concentrations present in S_LS, S_PS, and S_PS_C extracts, respectively; 5-CQA: 1.98 × 10^−3^ mg/mL, 1.04 × 10^−3^ mg/mL and 7.16 × 10^−4^ mg/mL, concentrations found in S_LS, S_PS, and S_PS_C extracts, respectively). The compounds were tested separately and combined.

The results on ^3^H-DG uptake suggest that caffeine and 5-CQA were not able to inhibit it by themselves (Figure 4g,h). These results are corroborated by those recently reported by Ontawong et al., where similar concentrations of these compounds were tested [45]. Nevertheless, there is also a study on 5-CQA showing that for higher concentrations (1 mM), this compound is able to inhibit the intestinal absorption of glucose by 80% [46], although such concentrations may hardly be present in this matrix. On the other hand, when combined with the same concentration found in each extract (Figure 4i (CAF+5-CQA)), a significant reduction in ^3^H-DG uptake was observed (by around 15% with all mixtures). This finding reinforces the results on ^3^H-DG uptake obtained with S_LS, S_PS, and S_PS_C extracts (Figure 4a–c), supporting the effectiveness of this combination. Nevertheless, although the reduction in ^3^H-DG uptake caused by caffeine+5-CQA mimicking the S_LS extract was rather similar to that found with the real extract, the reductions found for caffeine+5-CQA mimicking S_PS and S_PS_C extracts were quite distinct from those found with the respective extracts, mostly with the S_PS_C extract. Thus, while the synergism between caffeine and 5-CQA seems to be the main responsible for the ^3^H-DG uptake reductions caused by the S_LS extract, other bioactive compounds besides caffeine and 5-CQA (which also acted synergically) might also contribute to the inhibition of the intestinal uptake of glucose caused by S_PS and S_PS_C extracts. This evidences once again that although the original sample has been exactly the same, the different methodologies implemented to prepare the extracts significantly influenced the results. In fact, considering that S_PS_C extract was prepared aiming the saturation and concentration of bioactive compounds, which was not reflected on caffeine and CGAs contents of the freeze-dried extracts, it is possible that during its preparation (where moderately high temperatures might have been attained during the extended extraction and rotatory evaporation), CGA-derived compounds might have been formed, such as those previously mentioned in Section 3.1 (e.g., melanoidins and CGLs), leading to the significantly higher reductions found for cells treated with this extract.

Regarding the effect of caffeine and 5-CQA upon ^14^C-FRU uptake, the results suggest that caffeine in the concentration present in S_PS extract diminished ^14^C-FRU absorption by Caco-2 cells in 13% (Figure 4j). Moreover, 5-CQA in the concentrations present in S_PS and S_PS_C extracts also repressed the uptake of this sugar by 14–17% (Figure 4k). To the best of our knowledge, this is the first study that reports the effect of these two compounds on intestinal fructose absorption. Moreover, when combined in the same concentrations as existing in the three extracts, caffeine+5-CQA were able to reduce ^14^C-FRU uptake in Caco-2 cells (13–18%) (Figure 4l). These data suggest that, effectively, a synergic activity between these compounds exists and that this synergism is more evident for the S_LS extract. Furthermore, as observed with ^3^H-DG uptake, the synergism between these two compounds seems to be the main responsible for the reduction in ^14^C-FRU uptake observed with the S_LS extract (because caffeine and 5-CQA alone did not affect ^14^C-FRU uptake, but when combined, a parallelism with the effect of the S_LS extract was found). Notwithstanding, contrary to what was observed with ^3^H-DG uptake, S_PS and S_PS_C extracts presented a slightly lower effect on ^14^C-FRU uptake than their respective combinations of caffeine and 5-CQA, suggesting that other compounds present in these extracts may have negatively affected the effect of caffeine+5-CQA on ^14^C-FRU uptake. If that is the case, these compounds were probably not present in the S_LS extract, thus reinforcing once again the importance of the methodology applied to prepare the extracts.

### 3.4. Effect of the Different Extracts upon SGLT1, GLUT2, and GLUT5 mRNA Levels

**Figure 5:**
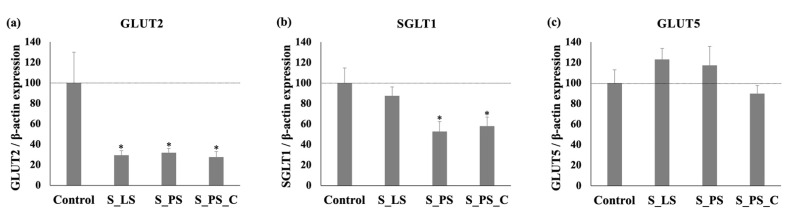
Quantification of mRNA levels of facilitative glucose transporter 2 (GLUT2) (**a**), sodium–glucose linked transporter 1 (SGLT1) (**b**), and facilitative glucose transporter 5 (GLUT5) (**c**), by qRT-PCR in Caco-2 cells after treatment for 24 h with 1 mg/mL of S_LS, S_PS, and S_PS_C extracts (*n* = 6 for each) or the respective solvent (control, DMSO; *n* = 5). Results are shown as the expression of SGLT1, GLUT5 or GLUT2 relative to β-actin (arithmetic means ± SEM). * Significantly different from control (*p* < 0.05).

In order to better characterize the effects of the extracts on intestinal sugar transport, the glucose and fructose intestinal transporters mRNA levels were quantified by qRT-PCR. Two facilitative glucose transporters (GLUT2 and GLUT5) and the sodium-glucose cotransporter (SGLT1) are responsible for the intestinal absorption of glucose and fructose: GLUT2 is capable of transporting both glucose and fructose, while GLUT5 is specific for fructose and SGLT1 transports only glucose [7]. As shown in Figure 5, a 24 h exposure of Caco-2 cells to S_LS, S_PS, and S_PS_C extracts resulted in a marked reduction in GLUT2 expression levels (to 29%, 32%, and 28% control, respectively) (Figure 5a). Regarding the SGLT1 extracts, S_PS and S_PS_C were able to induce a sharp decrease in this transporter mRNA levels (to 53% and 58% of control, respectively) (Figure 5b). However, none of these extracts were able to modify GLUT5 gene expression (Figure 5c). These results and, more particularly, the results for GLUT2 transporter, are very promising, since GLUT2 has been appointed as the most important pathway for the intestinal absorption of sugars when high doses of glucose and fructose are ingested, namely at the postprandial state, since the consumption of high-sugar foods is responsible for the rapid translocation of the GLUT2 transporter to the apical side of the membrane [7,47,48,49,50].

Moreover, by comparing the effect of the extracts concerning ^3^H-DG and ^14^C-FRU uptake assays with the qRT-PCR results, it can be verified that the noticeable reductions in gene expression levels of sugar transporters (mainly GLUT2, but also SGLT1) are not accompanied by similarly marked reductions in ^3^H-DG and ^14^C-FRU uptake. For example, while S_LS extract decreased the expression levels of GLUT2 and SGLT1 by 71% and 12%, respectively (Figure 5a,b), the same extract reduced by approximately 17% the absorption of ^3^H-DG (Figure 4a) and by 19% the absorption of ^14^C-FRU (Figure 4d). The disparity in the extent of the extracts’ effects on transporter activity and mRNA levels proposes that their effect on sugar uptake is not just the consequence of a modification in transcriptional levels of GLUT2 and SGLT1.

In fact, the activity of these transporters depends on the rates of transcription, translation (protein level), insertion of transporters into the membrane, intracellular signaling pathway and their intrinsic activity. For instance, gene methylation through epigenetic mechanisms [51,52] and mRNA stability [53] are already known to alter the activity of transporters. of GLUT. In this way, although the mRNA expression of GLUT2 and SGLT1 transporters has been markedly inhibited by the extracts, when analyzing the effect on the cells as a whole, that is, considering all steps from gene expression until transporters activity, the inhibitions of those expression genes were not so evident. Even so, these effects were quite significant and beneficial.

### 3.5. Effect of the Different Extracts on Cell Viability and Culture Mass

Considering the significant reductions in ^3^H-DG and ^14^C-FRU uptake and in GLUT2 and SGLT1 mRNA levels observed with all extracts, we decided to rule out the possibility that these effects were resultant from a cytotoxic action by determining their effects in cell viability and culture mass. At a concentration of 1 mg/mL, the extracts were not cytotoxic, as evaluated by the LDH assay (no significant differences between control and extracts, *p* > 0.05), although a small reduction (between 16 and 24%, *p* < 0.05) in culture mass was observed in the SRB assay.

Thus, we may conclude that the reductions found in the previous assays were not associated with a decrease in cell viability. Nevertheless, the reductions in culture mass found for all extracts through the SRB assay demonstrate that it might be possible that they present an antiproliferative effect. So, it would be interesting to further investigate these findings in order to add even more value to this coffee by-product.

## 4. Conclusions

Overall, these results indicate that silverskin might be a useful ingredient in the development of functional food products or dietary supplements and that UAE can be a viable option to be applied by industries for the recovery of the desired compounds from natural matrices.

Indeed, for coffee silverskin, no significant differences (*p* > 0.05) were found between the caffeine contents of the extracts prepared at the laboratory and pilot scales, and only minor differences were observed between the CGAs profiles. However, some CGAs degradation was noticed when one of the extracts prepared at the pilot scale was subjected to concentration in a rotary evaporator for a long period (8 h) at 40 °C, which highlights the perishability of these compounds and, thus, a limitation that must be addressed and solved in further studies in order to increase their concentration. Notwithstanding, the low amounts of compounds in this extract were not reflected in less beneficial effects on intestinal glucose and fructose uptake capacity by Caco-2 cells.

When testing the effect of the main compounds detected in the freeze-dried extracts (caffeine and 5-CQA) on glucose and fructose absorption, it was found that at the concentrations present in the three extracts, the isolated compounds have a low capacity to inhibit sugars uptake. However, when caffeine and 5-CQA are mixed, they present a synergistic effect. Even though, when compared to the effects of the extracts, this combination presented generally slightly different results, suggesting that other compounds may also interact with caffeine and 5-CQA. Therefore, in further studies, it would be interesting to better characterize these extracts regarding the presence of other bioactive compounds, such as other CGAs and CGA derivatives (e.g., melanoidins and CGA lactones), to evaluate their effects on the intestinal absorption of sugars and also to the find possible interactions (synergistic, antagonistic, and/or additive) between all compounds that might contribute to the observed effects.

Finally, it was found that the effects on glucose and fructose uptake resulted mainly from the inhibition of GLUT2 and SGLT1 genes expression but not from the inhibition of GLUT5 gene expression. Nonetheless, in further studies, it would be also interesting to investigate other effects on glucose and fructose transport besides the inhibition of the gene expressions of these transporters (e.g., their intrinsic activity), as well as other mechanisms of action of these extracts that may be relevant in the context of MetS. In addition, it would be also important to study these effects in vivo in order to better understand the real benefits they can have.

To conclude, in further studies, considering the existing literature on the possible incorporation of coffee silverskin and its extracts in foods and the effects found in this study on sugar uptake, it would be interesting to develop a functional food (e.g., a bread or a snack) aimed at preventing DM2 and other metabolic-related disorders.

## Figures and Tables

**Table 1:** Caffeine and chlorogenic acids contents (in mg/g of freeze-dried powder) of the different coffee silverskin extracts.

Extract	Caffeine	5-CQA	4-CQA	3-CQA	5-FQA	4-FQA
S_LS	27.73 ^a^ ± 0.16	1.98 ^a^ ± 0.05	0.15 ^b^ ± 0.04	0.31 ^a^ ± 0.09	1.32 ^a^ ± 0.05	0.43 ^a^ ± 0.04
S_PS	29.61 ^a^ ± 1.76	1.04 ^b^ ± 0.02	0.42 ^a^ ± 0.01	0.19 ^ab^ ± 0.04	1.05 ^ab^ ± 0.30	0.39 ^a^ ± 0.04
S_PS_C	27.33 ^a^ ±0.07	0.72 ^c^ ± 0.01	0.14 ^b^ ± 0.01	0.06 ^b^ ± 0.03	0.59 ^b^ ± 0.01	0.37 ^a^ ± 0.03

Results are expressed as means ± standard deviation. Within each column, different letters represent significant differences between samples at *p* < 0.05. CQA, caffeoylquinic acid; FQA, feruloylquinic acid. Note: S_LS (laboratorial scale) and S_PS (pilot scale) were directly freeze-dried after extraction; S_PS_C (pilot scale; concentrated extract) was subjected to rotatory evaporation (8 h, at 40 °C) and then freeze-dried.

## Data Availability

Not applicable.

## Abbreviations

^14^C-FRU	^14^C-D-Fructose
^3^H-DG	[1,2-^3^H(N)]-deoxy-D-glucose, ^3^H-deoxy-D-glucose
AT	Annealing temperature
cDNA	Complementary deoxyribonucleic acid
CGAs	Chlorogenic acids
CGLs	Chlorogenic acid lactones
CQA	Caffeoylquinic acid
DM2	Type 2 diabetes mellitus
DMSO	Dimethyl sulfoxide
DNA	Deoxyribonucleic acid
EDTA	Ethylenediamine tetra-acetic acid
FQA	Feruloylquinic acid
GLUT	Facilitative glucose transporter
HEPES	N-2-hydroxyethylpiperazine-N′-2-ethanesulfonic acid
HFCS	High-fructose corn syrup
HPLC	High-performance liquid chromatography
LDH	Lactate dehydrogrenase
MEM	Minimum essential medium
MetS	Metabolic syndrome
MMM	Multi-frequency multimode modulation
mRNA	Messenger ribonucleic acid
NADH	Nicotinamide adenine dinucleotide
qRT-PCR	Quantitative reverse transcription polymerase chain reaction
RP-HPLC-DAD	Reverse-phase high-performance liquid chromatography coupled to diode array detector
RNA	Ribonucleic acid
SEM	Standard error of the mean
SGLT1	Sodium-glucose linked transporter 1
SRB	Sulforhodamine B
S_LS	Silverskin extract prepared at laboratorial scale
S_PS	Silverskin extract prepared at pilot scale
S_PS_C	Concentrated silverskin extract prepared at pilot scale
UAE	Ultrasound-assisted extraction

## References

[1] Bessada S.M.F., Alves R.C., Costa A.S.G., Nunes M.A., Oliveira M.B.P.P. (2018). Coffea canephora silverskin from different geographical origins: A comparative study. Sci. Total Environ..

[2] Mussatto S.I., Machado E.M.S., Martins S., Teixeira J.A. (2011). Production, composition, and application of coffee and its industrial residues. Food Bioprocess Technol..

[3] Puga H., Alves R.C., Costa A.S., Vinha A.F., Oliveira M.B.P.P. (2017). Multi-frequency multimode modulated technology as a clean, fast, and sustainable process to recover antioxidants from a coffee by-product. J. Clean. Prod..

[4] Costa A.S.G., Alves R.C., Vinha A.F., Costa E., Costa C.S.G., Nunes M.A., Almeida A.A., Santos-Silva A., Oliveira M.B.P.P. (2018). Nutritional, chemical and antioxidant/pro-oxidant profiles of silverskin, a coffee roasting by-product. Food Chem..

[5] Nzekoue F.K., Borsetta G., Navarini L., Abouelenein D., Xiao J., Sagratini G., Vittori S., Caprioli G., Angeloni S. (2022). Coffee silverskin: Characterization of B-vitamins, macronutrients, minerals and phytosterols. Food Chem..

[6] de Melo Pereira G.V., de Carvalho Neto D.P., Magalhães Júnior A.I., do Prado F.G., Pagnoncelli M.G.B., Karp S.G., Soccol C.R. (2020). Chemical composition and health properties of coffee and coffee by-products. Adv. Food Nutr. Res..

[7] Loureiro G., Martel F. (2019). The effect of dietary polyphenols on intestinal absorption of glucose and fructose: Relation with obesity and type 2 diabetes. Food Rev. Int..

[8] Pimentel G.D., Micheletti T.O., Fernandes R.C., Nehlig A., Watson R. (2019). Coffee intake and obesity. Nutrition in the Prevention and Treatment of Abdominal Obesity.

[9] Tunnicliffe J.M., Cowan T., Shearer J., Preedy V.R. (2015). Chlorogenic acid in whole body and tissue-specific glucose regulation. Coffee in Health and Disease Prevention.

[10] Meng T., Antony B., Venn A., Fraser B., Cicuttini F., March L., Cross M., Dwyer T., Jones G., Laslett L.L. (2020). Association of glucose homeostasis and metabolic syndrome with knee cartilage defects and cartilage volume in young adults. Semin. Arthritis Rheum..

[11] De Oliveira D.T., Fernandes I.D.C., De Sousa G.G., Dos Santos T.A.P., De Paiva N.C.N., Carneiro C.M., Evangelista E.A., Barboza N.R., Guerra-Sá R. (2020). High-sugar diet leads to obesity and metabolic diseases in ad libitum-fed rats irrespective of caloric intake. Arch. Endocrinol. Metab..

[12] Seo E.H., Kim H., Kwon O. (2019). Association between total sugar intake and metabolic syndrome in middle-aged Korean men and women. Nutrients.

[13] Bullo M., Cozar-Torrell P., Salas-Salvado J. (2014). Dietary regulation of glucose metabolism in metabolic syndrome. Curr. Vasc. Pharmacol..

[14] Hosseini-Esfahani F., Bahadoran Z., Mirmiran P., Hosseinpour-Niazi S., Hosseinpanah F., Azizi F. (2011). Dietary fructose and risk of metabolic syndrome in adults: Tehran lipid and glucose study. Nutr. Metab..

[15] Taskinen M.R., Packard C.J., Borén J. (2019). Dietary fructose and the metabolic syndrome. Nutrients.

[16] Guarino M.P., Sacramento J., Ribeiro M.J., Conde S.V., Preedy V.R. (2015). Caffeine, insulin resistance, and hypertension. Coffee in Health and Disease Prevention.

[17] Johnston K.L., Clifford M.N., Morgan L.M. (2003). Coffee acutely modifies gastrointestinal hormone secretion and glucose tolerance in humans: Glycemic effects of chlorogenic acid and caffeine. Am. J. Clin. Nutr..

[18] Lane J.D. (2011). Caffeine, glucose metabolism, and type 2 diabetes. J. Caf. Res..

[19] Robertson T.M., Clifford M.N., Penson S., Williams P., Robertson M.D. (2018). Postprandial glycaemic and lipaemic responses to chronic coffee consumption may be modulated by CYP1A2 polymorphisms. Br. J. Nutr..

[20] Alves R.C., Casal S., Oliveira B. (2009). Benefícios do café na saúde: Mito ou realidade?. Quím. Nova.

[21] Baspinar B., Eskici G., Ozcelik A.O. (2017). How coffee affects metabolic syndrome and its components. Food Funct..

[22] Ludwig I.A., Clifford M.N., Lean M.E.J., Ashihara H., Crozier A. (2014). Coffee: Biochemistry and potential impact on health. Food Funct..

[23] Bondam A.F., da Silveira D.D., dos Santos J.P., Hoffmann J.F. (2022). Phenolic compounds from coffee by-products: Extraction and application in the food and pharmaceutical industries. Trends Food Sci. Technol..

[24] Iriondo-De Hond A., Iriondo-De Hond M., del Castillo M.D. (2020). Applications of compounds from coffee processing by-products. Biomolecules.

[25] Torres-Valenzuela L.S., Ballesteros-Gómez A., Rubio S. (2020). Green solvents for the extraction of high added-value compounds from agri-food waste. Food Eng. Rev..

[26] Chemat F., Rombaut N., Sicaire A.-G., Meullemiestre A., Fabiano-Tixier A.-S., Abert-Vian M. (2017). Ultrasound assisted extraction of food and natural products. Mechanisms, techniques, combinations, protocols and applications. A review. Ultrason. Sonochem..

[27] Schmittgen T.D., Livak K.J. (2008). Analyzing real-time PCR data by the comparative CT method. Nat. Prot..

[28] Andrade N., Silva C., Martel F. (2018). The effect of oxidative stress upon intestinal sugar transport: An in vitro study using human intestinal epithelial (Caco-2) cells. Toxicol. Res..

[29] Bradford M.M. (1976). A rapid and sensitive method for the quantitation of microgram quantities of protein utilizing the principle of protein-dye binding. Anal. Biochem..

[30] Medina-Torres N., Ayora-Talavera T., Espinosa-Andrews H., Sánchez-Contreras A., Pacheco N. (2017). Ultrasound assisted extraction for the recovery of phenolic compounds from vegetable sources. Agronomy.

[31] Vilkhu K., Mawson R., Simons L., Bates D. (2008). Applications and opportunities for ultrasound assisted extraction in the food industry—A review. Innov. Food Sci. Emerg. Technol..

[32] Costa A.S.G., Alves R.C., Vinha A.F., Barreira S.V.P., Nunes M.A., Cunha L.M., Oliveira M.B.P.P. (2014). Optimization of antioxidants extraction from coffee silverskin, a roasting by-product, having in view a sustainable process. Ind. Crops Prod..

[33] Narita Y., Inouye K. (2012). High antioxidant activity of coffee silverskin extracts obtained by the treatment of coffee silverskin with subcritical water. Food Chem..

[34] Narita Y., Inouye K., Preedy V.R. (2015). Chlorogenic acids from coffee. Coffee in Health and Disease Prevention.

[35] Wianowska D., Gil M. (2019). Recent advances in extraction and analysis procedures of natural chlorogenic acids. Phytochem. Rev..

[36] Farah A., de Paulis T., Moreira D.P., Trugo L.C., Martin P.R. (2006). Chlorogenic acids and lactones in regular and water-decaffeinated arabica coffees. J. Agric. Food Chem..

[37] Alves R.C., Rodrigues F., Nunes M.A., Vinha A.F., Oliveira M.B.P.P., Galanakis C.M. (2017). State of the art in coffee processing by-products. Handbook of Coffee Processing By-Products.

[38] Habtemariam S., Habtemariam S. (2019). Chemical and pharmacological evidences for coffee as a modulator of type 2 diabetes and metabolic syndrome. Medicinal Foods as Potential Therapies for Type-2 Diabetes and Associated Diseases–The Chemical and Pharmacological Basis of Their Action.

[39] Shearer J., Farah A., de Paulis T., Bracy D.P., Pencek R.R., Graham T.E., Wasserman D.H. (2003). Quinides of roasted coffee enhance insulin action in conscious rats. J. Nutr..

[40] Rufián-Henares J.A., Pastoriza S., Preedy V.R. (2015). Melanoidins in coffee. Coffee in Health and Disease Prevention.

[41] De la Cruz S.T., Iriondo-DeHond A., Herrera T., Lopez-Tofiño Y., Galvez-Robleño C., Prodanov M., Velazquez-Escobar F., Abalo R., del Castillo M.D. (2019). An assessment of the bioactivity of coffee silverskin melanoidins. Foods.

[42] Pérez-Burillo S., Rajakaruna S., Pastoriza S., Paliy O., Rufián-Henares J.A. (2020). Bioactivity of food melanoidins is mediated by gut microbiota. Food Chem..

[43] Iriondo-DeHond A., Fernandez-Gomez B., Martinez-Saez N., Martirosyan D.M., Garcia M.D.M., del Castillo M.D. (2017). Coffee silverskin: A low-cost substracte for bioproduction of high-value health promoting products. Ann. Nutr. Food Sci..

[44] Nunes M.A., Reszczynski F., Páscoa R.N.M.J., Costa A.S.G., Alves R.C., Oliveira M.B.P.P. (2021). Influence of olive pomace blending on antioxidant activity: Additive, synergistic, and antagonistic effects. Molecules.

[45] Ontawong A., Duangjai A., Srimaroeng C. (2021). Coffea arabica bean extract inhibits glucose transport and disaccharidase activity in Caco-2 cells. Biomed. Rep..

[46] Welsch C.A., Lachance P.A., Wasserman B.P. (1989). Dietary phenolic compounds: Inhibition of Na^+^-dependent D-glucose uptake in rat intestinal brush border membrane vesicles. J. Nutr..

[47] Andrade N., Araújo J.R., Correia-Branco A., Carletti J.V., Martel F. (2017). Effect of dietary polyphenols on fructose uptake by human intestinal epithelial (Caco-2) cells. J. Funct. Foods.

[48] Douard V., Ferraris R.P. (2008). Regulation of the fructose transporter GLUT5 in health and disease. Am. J. Physiol. Endocrinol. Metab..

[49] Kellett G.L., Brot-Laroche E. (2005). Apical GLUT2: A major pathway of intestinal sugar absorption. Diabetes.

[50] Kellett G.L., Brot-Laroche E., Mace O.J., Leturque A. (2008). Sugar absorption in the intestine: The role of GLUT2. Ann. Rev. Nutr..

[51] Novakovic B., Gordon L., Robinson W.P., Desoye G., Saffery R. (2013). Glucose as a fetal nutrient: Dynamic regulation of several glucose transporter genes by DNA methylation in the human placenta across gestation. J. Nutr. Biochem..

[52] Shanak S., Saad B., Zaid H. (2019). Metabolic and epigenetic action mechanisms of antidiabetic medicinal Plants. Evid. Based Complement. Alternat. Med..

[53] Gouyon F., Caillaud L., Carrière V., Klein C., Dalet V., Citadelle D., Kellett G.L., Thorens B., Leturque A., Brot-Laroche E. (2003). Simple-sugar meals target GLUT2 at enterocyte apical membranes to improve sugar absorption: A study in GLUT2-null mice. J. Physiol..

